# Large outbreak of tularaemia, central Sweden, July to September 2019

**DOI:** 10.2807/1560-7917.ES.2019.24.42.1900603

**Published:** 2019-10-17

**Authors:** Rikard Dryselius, Marika Hjertqvist, Signar Mäkitalo, Anders Lindblom, Tobias Lilja, Disa Eklöf, Anders Lindström

**Affiliations:** 1 Public Health Agency of Sweden, Solna, Sweden; 2 Gävleborg County Council, Gävle, Sweden; 3 Dalarna County Council, Falun, Sweden; 4 National Veterinary Institute, Uppsala, Sweden

**Keywords:** tularaemia, outbreak, Francisella tularensis, mosquito bites, vector borne disease, Sweden

## Abstract

On 31 of July 2019, the Public Health Agency of Sweden was alerted about an increasing number of tularaemia cases in Gävleborg, a county in central Sweden. The number of cases increased thereafter peaking at about 150 reports of illnesses every week. As at 6 October, a total of 979 cases (734 laboratory-confirmed) have been reported, mainly from counties in central Sweden. The outbreak is now considered over (as at 14 October).

In 2019, Sweden is experiencing its largest outbreak of tularaemia in over 50 years. The outbreak started in July and as at 6 October 2019, a total of 979 cases have been reported. Here, we report the demographic features of cases and geographical distribution of the outbreak. We also provide evidence that the predominant route of transmission is via mosquito bites and want to make visitors aware of risks in endemic areas.

## Identification of the outbreak

In the last week of July 2019, the County Medical Officer (CMO) of Gävleborg county, central Sweden, received information from healthcare providers about an increasing number of people visiting healthcare centres with fever and presenting swollen inguinal and axillary lymph nodes. By 31 July, there were 18 reported cases of tularaemia, all were residents of or visitors to a small town in the county, Ljusdal. According to healthcare providers in Ljusdal, a majority had spent time near a lake and a golf course adjacent to the town. As a major golf competition with visitors from all over Sweden had been held between 23-27 July, the CMO issued a press release on 31 July announcing the outbreak and put information on the Swedish national healthcare website (1177 Vårdguiden) to alert health care in regions not used to diagnosing the disease.

On 2 August, the Swedish National Veterinary Institute (SVA) published a news item about a suspected outbreak of tularaemia among hares (*Lepus* species) as a large number of dead hares had been detected and that tularaemia had been identified in hares from several locations around Sweden.

During the first week of August, an unusually large number of human tularaemia cases were reported to the Public Health Agency of Sweden (PHAS) from other locations, mainly in neighbouring counties to Gävleborg in central Sweden. On 12 August, the PHAS published a news item on its website about the outbreak. At that time, the number of reported tularaemia cases had reached over 80 in the county of Dalarna where a major cycling competition with more than 20,000 national and international participants was taking place between 10 and 16 August. A case definition for tularaemia can be seen in Box.

Box **Tularaemia case definition, Sweden**

**Confirmed case:** At least one of the following three:

• Isolation of *Francisella tularensis*

• Detection of *Francisella tularensis* nucleic acid

• *Francisella tularensis* specific antibody response

**Probable case:** Both of the following two:

• Clinical picture compatible with tularaemia

• Epidemiological link

## Descriptive epidemiology

**Figure 1:**
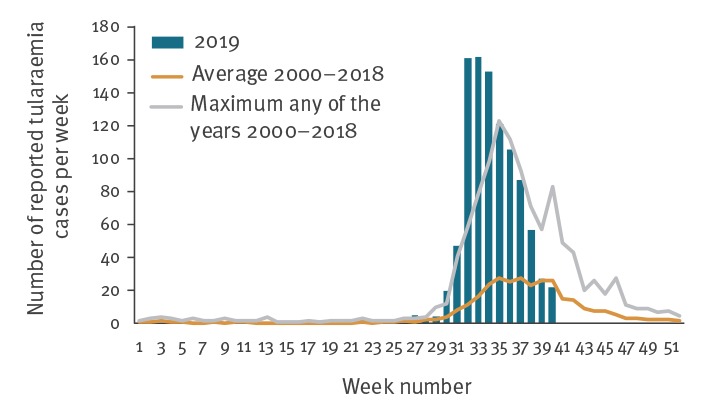
Number of reported tularaemia cases per week, Sweden, 2019 and as an average between 2000–2018

As at week 40 2019 (6 October), a total of 979 tularaemia cases were reported to PHAS, 98% of which were registered since 24 July – over four times more than the average number for the corresponding periods between 2000 and 2018 (Figure 1). Of the reported cases, 734 were laboratory-confirmed mainly based on serology (n = 404) or PCR (n = 285).

**Figure 2:**
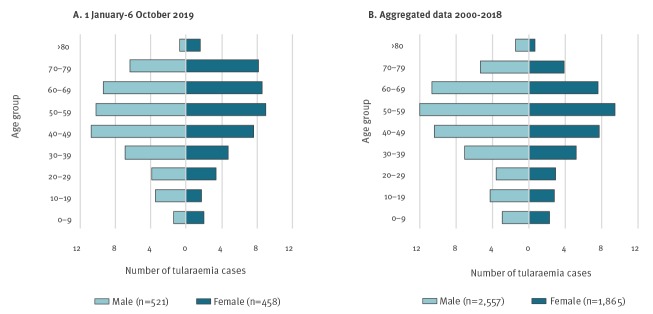
Age and sex distribution of tularaemia cases (A) 1 January–6 October 2019 (n = 979) and (B) aggregated data from the same period in 2000–2018 (n = 4,422), Sweden

Of 979 reported cases in 2019, 521 (53%) were male and 458 (47%) female. The median age was 52 years and higher for females than males (55 vs 50 years, respectively) (Figure 2).

Between 1 January and 6 October each year the period 2000-18, 4,422 tularaemia cases were reported of which, 2,557 (58%) were male and 1,865 (42%) female with median ages of 50 years for both sexes. A significantly larger proportion of females above the age of 50 years were infected with tularaemia in 2019 compared with the period between 2000 and 2018 (chi-squared test: p < 0.001).

**Figure 3:**
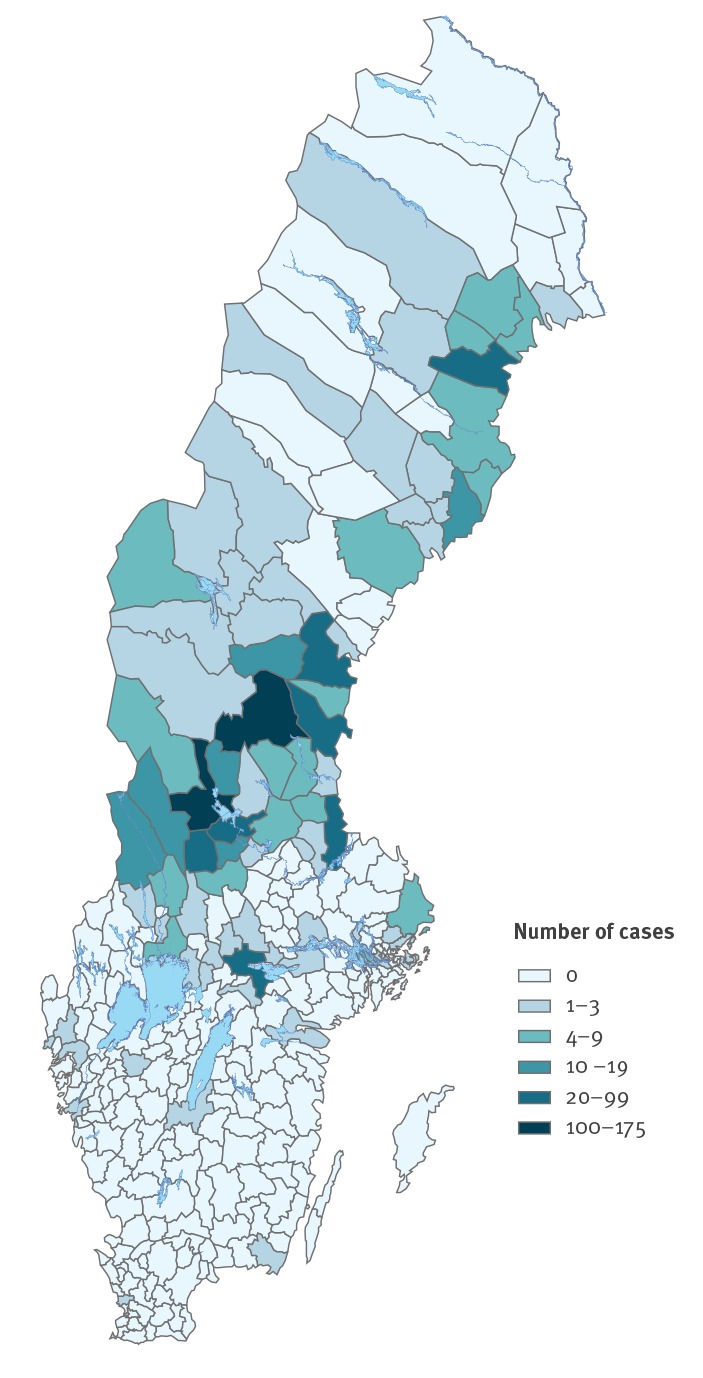
Number of tularaemia cases by municipality, Sweden, 1 January–6 October 2019

For 752 of 979 tularaemia cases in 2019, a specific municipality could be designated as the probable place of infection based on the information given in clinical reports. Fifteen municipalities reported 10 or more cases and 13 were located in central Sweden. In two municipalities over 100 cases were reported (Figure 3).

Dalarna county was the most affected by the tularaemia outbreak and according to reports from healthcare centres 268 of 326 cases (82%) had the ulceroglandular form of tularaemia; information was missing for 58 cases. A similar dominance of ulceroglandular tularaemia has also been reported from local healthcare centres in Gävleborg county with patients having inflamed and sometimes infected mosquito bites. There have also been four reports of pneumonic cases.

Of 979 tularaemia cases reported nationally until 6 October 2019, 719 (73%) had a cause of infection specified as insect bites, which is higher than the corresponding period 2000–18 (2,729/4,422 cases; 62%).

## Environmental investigation and findings in wildlife

Mosquitoes were collected around the golf course in Ljusdal on 15 and 16 August 2019. A total of 550 mosquitoes were trapped and of those 373 (68%) belonged to the known tularaemia vector *Aedes cinereus* (Table 1). All other species found, except *Culiseta morsitans*, have previously been shown to be implicated in tularaemia transmission [1]. Mosquitoes were divided into species pools based on their trapping location; in total, there were 24 pools. Molecular analyses showed that one of eight pools of *Aedes cinereus* containing 103 specimens that were collected near the tee to hole 14 and known to be a particularly mosquito-infested area among golfers, tested positive for *Francisella tularensis holarctica*.

**Table 1:** Species and number of mosquitoes collected around a golf course, Ljusdal, Sweden, 15 and 16 August 2019 (n = 550)

Mosquito species	Number of mosquitoes
*Aedes cinereus*	373
*Aedes* sp.	73
*Aedes cantans/annulipes*	65
*Aedes sticticus*	33
*Aedes communis*	4
*Culiseta morsitans*	1
*Anopheles maculipennis s.l.*	1

**Figure 4:**
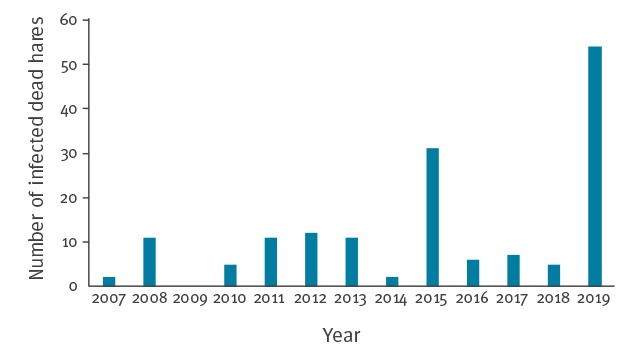
Number of dead hares with tularaemia submitted to the Swedish National Veterinary Institute, Sweden, 2007–2019 (n = 157)

In Sweden, dead hares are routinely collected and analysed by the SVA [2]. In 2019, 54 dead hares with tularaemia were collected, while the mean annual number of dead hares with tularaemia between 2007 and 2018 was nine (Figure 4).

Data reported until 30 September 2019.

## Discussion and conclusion

Tularaemia is caused by the highly contagious bacterium *Francisella tularensis*, which can infect humans as well as more than 250 animal species [3]. Tularaemia is widely spread throughout the northern hemisphere and cases have been reported from all European countries except Iceland, Ireland and the United Kingdom [4]. From some parts of Sweden and Finland, where tularaemia occurs endemically, the highest incidence in Europe is reported [5].

Sweden has reported cases of endemic tularaemia since 1931 [6]. There has always been a variation in the number of human cases between different years, ranging from 0 cases in 1990 to 2,700 cases in 1967. During the 2000s, the number of reported cases varied from 26 in 2001 to 859 in 2015, with a resulting incidence of 0.29 and 8.72 cases per 100,000 inhabitants, respectively. However, no cyclical patterns or general trends were observed (personal communication Pontus Juréen, PHAS, 30 Sep 2019). The number of reported tularaemia cases thus far in 2019 is higher than what has been reported in any single year since the end of the 1960s and it is more than are reported from the whole of Europe during a normal year [4].

Historically, more males were reported with tularaemia infection than females, with the age group 40–69 years most affected for both sexes; this pattern is seen in outbreak years as well years with a low number of reported infections (data not shown). This may be a reflection of the age distribution of people in rural areas working outdoors e.g. doing farm and garden work [7]. In the current outbreak, a larger proportion of women aged 50 years and older has been observed but the reasons for this deviating pattern is unknown. It is possible, for example, that with 2019 being a good mushroom year, more women in this age group were attracted to tularaemia-risk areas to pick mushrooms.

Ever since the first tularaemia cases were reported in 1931 until the early 1990s, the endemic area was in the northern part of central Sweden and extended along the Gulf of Bothnia [8]. Since the end of the 1990s, the geographical distribution of cases has expanded with a spread both to the west and towards the southern parts of central Sweden [9]. This expanded area has since remained endemic for tularaemia and experienced larger outbreaks in its northern part in 2012 and 2015, in its central part in 2010 and in its southern part in 2000 and 2003. An expansion of the endemic area further south is not unlikely in the future – supported by the numerous dead hares with tularaemia detected up to September 2019 by SVA, where at least eight had been found in Götaland located south of the endemic area [10].

Tularaemia can be transmitted to humans through arthropod vectors, direct contact with infected animals or by inhalation of contaminated dust. Due to the mode of transmission, different clinical forms are distinguished. In neighbouring Norway and elsewhere in Europe, ingestion of contaminated water from streams, ponds, lakes and rivers is the main mode of infection [4,11]. In Sweden, however, most cases are infected by mosquito bites, but transmission by ticks and rain flies also occurs [12]. Typically, mosquito-transmitted ulceroglandular tularaemia starts to become evident late July or August, reaching a peak at the end of August or in September before declining up to December. Large mosquito populations are a risk factor for outbreaks of tularaemia [13]. In this outbreak, the time of onset of symptoms in cases e.g. reports of ulceroglandular tularaemia plus inflamed or infected mosquito bites, suggests that the predominant mode of transmission was via mosquitoes. This is supported by the detection of *Francisella tularensis* in the mosquito species *Aedes cinereus* near the golf course in Ljusdal. The weather conditions in 2019 with a relatively wet spring and a mild summer and autumn may have resulted in a favourable year for mosquito populations, meaning that transmission of tularaemia between mosquitoes and their host animals may have been longer and more favourable than normal. However, it was not possible to investigate this further, as mosquito densities in high-risk areas are not monitored nor are the populations of potential animal hosts. Interestingly, however, the outbreaks of tularaemia in central Sweden in 2003 and 2010 and in northern Sweden in 2012 and 2015 were all preceded by wetter springs than normal in the high-risk areas.

As at 14 October, the number of new cases of tularaemia has dropped drastically returning to average seasonal levels for the previous 3 weeks. However, due to the unusually high number of cases concentrated to areas with large numbers of external visitors, the 2019 tularaemia outbreak has prompted intense communication between local and central authorities and attracted a great deal of media attention in Sweden. With this rapid communication, we aim to raise awareness for the risk of contracting tularaemia through mosquito bites in endemic areas in Sweden and to raise awareness regarding protective measures such as wearing covering clothing and use of insect repellent. We also aim to raise awareness of tularaemia among health care providers in non-endemic areas, as they may encounter patients who have acquired tularaemia infection visiting high-risk areas such as in central and northern Sweden.

## References

[1] Kampen H, Walther D. Vector potential of mosquito species (Diptera: Culicidae) occurring in central Europe. Mosquito-borne Diseases. Implications for public health. Parasitology Research Monographs 10. Springer. Eds: Benelli, G. and Mehlhorn, H. Springer International Publishing. 2018.

[2] Statens Veterinärmedicinska anstalt (SVA). Surveillance of infectious diseses in animials and humans in Sweden. Uppsala: SVA; [accessed 7 Oct 2019]. Swedish. Available from: https://www.sva.se/om-sva/publikationer/sjukdomsovervakning/rapport-surveillance-of-infectious-diseases

[3] Mörner T The ecology of tularaemia. Rev Sci Tech. 1992;11(4):1123-30. 10.20506/rst.11.4.657 1305858

[4] European Centre for Disease Prevention and Control (ECDC). Tularaemia. Factsheet. Stockholm: ECDC; [Accessed 7 Oct 2019]. Available from: https://ecdc.europa.eu/en/tularaemia/facts

[5] Cross AR Baldwin VM Roy S Essex-Lopresti AE Prior JL Harmer NJ Zoonoses under our noses. Microbes Infect. 2019;21(1):10-9. 10.1016/j.micinf.2018.06.001 29913297 PMC6386771

[6] Payne L Arneborn M Tegnell A Giesecke J Endemic tularemia, Sweden, 2003. Emerg Infect Dis. 2005;11(9):1440-2. 10.3201/eid1109.041189 16229776 PMC3310613

[7] Hjertqvist M Ahlm C Klingström J Sex patterns in diagnoses of tularaemia, Sweden 1997-2008. J Infect. 2010;60(2):186-7. 10.1016/j.jinf.2009.11.012 20004687

[8] Tärnvik A Sandström G Sjöstedt A Epidemiological analysis of tularemia in Sweden 1931-1993. FEMS Immunol Med Microbiol. 1996;13(3):201-4. 10.1111/j.1574-695X.1996.tb00237.x 8861029

[9] Hjertqvist M, Arneborn M, Bäck E. Emergence of tularaemia in Sweden - an epidemiological survey of the last 35 years. 2006. Master thesis in Communicable Diseases and Infection Control.

[10] Statens Veterinärmedicinska anstalt (SVA). Karta över harpest. Uppsala: SVA; [Accessed 14 Oct 2019]. Swedish. Available from: https://www.sva.se/smittlage/karta-over-harpest

[11] Larssen KW Bergh K Heier BT Vold L Afset JE All-time high tularaemia incidence in Norway in 2011: report from the national surveillance. Eur J Clin Microbiol Infect Dis. 2014;33(11):1919-26. 10.1007/s10096-014-2163-2 24874046

[12] Eliasson H Broman T Forsman M Bäck E Tularemia: current epidemiology and disease management. Infect Dis Clin North Am. 2006;20(2):289-311, ix. 10.1016/j.idc.2006.03.002 16762740

[13] Rydén P Björk R Schäfer ML Lundström JO Petersén B Lindblom A Outbreaks of tularemia in a boreal forest region depends on mosquito prevalence. J Infect Dis. 2012;205(2):297-304. 10.1093/infdis/jir732 22124130 PMC3244368

